# Central conducting lymphatic anomaly: from bench to bedside

**DOI:** 10.1172/JCI172839

**Published:** 2024-04-15

**Authors:** Luciana Daniela Garlisi Torales, Benjamin A. Sempowski, Georgia L. Krikorian, Kristina M. Woodis, Scott M. Paulissen, Christopher L. Smith, Sarah E. Sheppard

**Affiliations:** 1 Unit on Vascular Malformations, Division of Intramural Research, *Eunice Kennedy Shriver* National Institute of Child Health and Human Development, NIH, Bethesda, Maryland, USA.; 2 Division of Cardiology, Jill and Mark Fishman Center for Lymphatic Disorders, Children’s Hospital of Philadelphia, Philadelphia, Pennsylvania, USA.

## Abstract

Central conducting lymphatic anomaly (CCLA) is a complex lymphatic anomaly characterized by abnormalities of the central lymphatics and may present with nonimmune fetal hydrops, chylothorax, chylous ascites, or lymphedema. CCLA has historically been difficult to diagnose and treat; however, recent advances in imaging, such as dynamic contrast magnetic resonance lymphangiography, and in genomics, such as deep sequencing and utilization of cell-free DNA, have improved diagnosis and refined both genotype and phenotype. Furthermore, in vitro and in vivo models have confirmed genetic causes of CCLA, defined the underlying pathogenesis, and facilitated personalized medicine to improve outcomes. Basic, translational, and clinical science are essential for a bedside-to-bench and back approach for CCLA.

## Introduction

### The lymphatic system and its functions.

**Figure 1:**
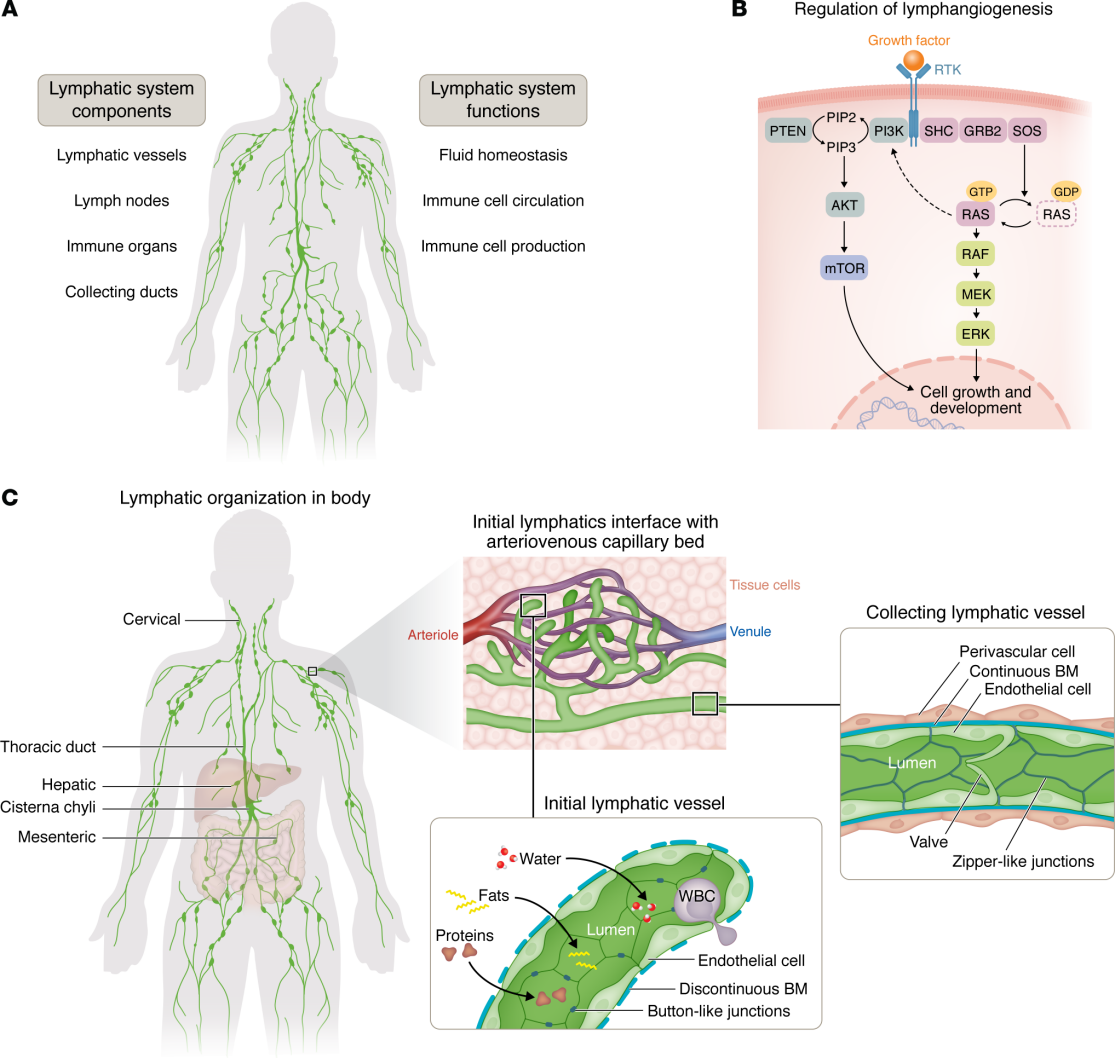
Overview of the lymphatic system and lymphangiogenesis. (**A**) Components and functions of the lymphatic system. (**B**) Schematic showing the major growth factor signaling pathways. Pathogenic variants in receptor tyrosine kinases, components of PI3K signaling, and components of RAS/MAPK signaling are important drivers of vascular anomalies. (**C**) Schematic showing the lymphatic system at the vascular level with important structures and interactions with the venous and arterial systems.

The lymphatic system is a network of vessels and associated organs that play an essential role in human health by maintaining human tissue fluid homeostasis, transporting immune cells, and absorbing dietary lipids (Figure 1A) (1). Lymphatic vessels transport interstitial fluid reabsorbed from tissues along with antigens and leukocytes destined for the lymph nodes that are then transferred to the blood. Lymphatic vasculature plays an important role in the gastrointestinal system, where peripheral lymphatics transport chyle, a mixture of lymph and chylomicrons broken down from dietary lipids to the thoracic duct (1–3). The meninges of the central nervous system also contain a vast network of lymphatic vessels that participate in the transportation of cerebrospinal fluid out of the brain (4, 5). In cases of human disease, the lymphatic system plays essential roles in regulating tissue pressure, immune surveillance, and energy regulation and has been implicated in chronic inflammation and cancer (1–3).

Molecular studies have demonstrated the role of specific signaling pathways in lymphatic development (Figure 1B). VEGF-C and its receptor VEGFR3 (also known as FLT4) are essential for lymphangiogenesis, the sprouting growth of lymphatic vessels. VEGFC signaling requires collagen- and calcium-binding EGF domains 1 (CCBE1) and ADAMTS3 for proteolytic cleavage (6–10). VEGFC/VEGFR3 signaling occurs through Erk to induce cell-cycle arrest and regulate lymphangiogenesis (11, 12). Molecular programs also direct the formation and maintenance of lymphatic valves — many of which also have been implicated in human disease. For example, FOXC2 along with flow activates connexin-37 (CX37) and calcineurin/NFATC signaling (13). GATA2, integrin-alpha9, ephrinB2/Ephb4 signaling, VE-cadherin, Foxo1, and Piezo1 all regulate valve formation and maintenance (14–23). Furthermore, disease models inducing overactivation of Ras signaling demonstrate valvular dysfunction (24).

The main structural components of the lymphatic system are lymphatic capillaries, lymphatic afferent and efferent vessels, lymph nodes, and lymphoid organs including the tonsils, thymus, spleen, and bone marrow (25). Lymphatic endothelial cells (LECs) constitute the most basic level of the lymphatic system and form the core lymphatic structures. LECs have both venous and nonvenous origins. They initially emerge from embryonic veins and are subsequently organized into lymphatic structures (6, 26–33). Among LECs, those of venous origin are thought to be the major contributors to the lymphatic vasculature (30–38). However, nonvenous origins have been demonstrated in organ-specific lymphatics, such as the mesentery (39), heart (40), and skin (41), including hemogenic endothelium (derived from the blood vasculature) contributing to dermal lymphatics (42). Lymphatic capillaries form interfaces with arteriovenous capillary beds throughout the body in a peripheral plexus (Figure 1C). This plexus is characterized by LECs with button junctions and a discontinuous basement membrane (BM) and blind-ended sacs that allow for the exchange of lipids, proteins, and water, as well as pathogens and immune cells. Lymphatic capillaries drain into precollecting lymphatics, then collecting lymphatic vessels, to the thoracic duct and the right lymphatic duct, and finally to the venous system. The collecting lymphatics are lined with endothelial cells that have zipper-like junctions and are encased in perivascular smooth muscle cells that contract to propel lymph forward and valves to prevent backflow of lymph. In humans, the central lymphatics include the cisterna chyli — where the lower extremity peripheral lymphatics and intra-abdominal lymphatics converge to a central drainage channel — and the thoracic duct (43). Disorders involving the central lymphatics may be seen in complex lymphatic anomalies (3, 44–49).

### Complex lymphatic anomalies.

Complex lymphatic anomalies (CLAs) are a group of overlapping disorders of diffuse lymphatic malformations caused by pathogenic variants (previously known as genetic mutations) (3, 44–48). These include central conducting lymphatic anomaly (CCLA), which also can be known as channel type lymphatic malformation, generalized lymphatic anomaly (GLA), kaposiform lymphangiomatosis (KLA), and Gorham-Stout disease (GSD). CCLA is a heterogenous disorder characterized by abnormal conduction through the central lymphatics (46). GLA is characterized by the invasion of lymphatic vessels into organs, such as the spleen, medullary bone, and the surrounding soft tissue due to the abnormal proliferation of LECs (49). KLA has some phenotypic overlap with GLA but is defined by the unique spindle-shaped morphology of affected LECs on biopsy (49–51). Mediastinal involvement as well as pericardial effusions may also be prevalent (52). KLA has a high morbidity due to the severity of the lesions (53). GSD, also known as vanishing bone disease, involves the spontaneous reabsorption of cortical bone due to the hyperproliferation of surrounding lymphatic vessels (54–56). It is important to note that all CLAs may have a degree of abnormal central lymphatic conduction (57–60).

### Central conducting lymphatic anomaly.

CCLA is one of the most heterogeneous types of CLA, as it encompasses any anomaly or dysfunction affecting the central lymphatic channels (thoracic duct and/or the cisterna chyli), which may become dilated, tortuous, or leaky. CCLA can be further classified as primary, with lymphatic dysfunction identified as the primary malformation, sometimes due to an identifiable genetic cause; or secondary, with lymphatic dysfunction acquired due to high central venous pressures or traumatic leaks (61, 62). Individuals affected by CCLA may present with nonimmune fetal hydrops, pleural effusions, ascites, anasarca, protein-losing enteropathy, plastic bronchitis, pulmonary lymphatic perfusion, and even lymphedema (44, 61, 63–65). These symptoms can be severely debilitating and even lead to death.

CCLA lacks concrete diagnostic criteria, and there is limited availability of central lymphatic imaging, which leaves some affected individuals with a symptomatic diagnosis (e.g., chylous ascites). CCLA can be found as a feature of multiple syndromes as well as an isolated disorder due to somatic genetic causes. Further research is needed to differentiate whether the genetic causes associated with CCLA (single-gene syndromes, mosaic somatic pathogenic mutations, aneuploidy, etc.) have disparate or similar underlying pathogenic mechanisms.

There is a profound need for continued basic and translational investigations to improve the lives of individuals with CCLA. The goal of this Review is to summarize the basic science that has led to clinical advances in CCLA. To do this, we first highlight the advances in imaging and molecular genetics that have resulted in improved diagnosis of CCLA. Next, we review the in vitro and in vivo models that have advanced our understanding of the cellular and molecular mechanisms underlying CCLA and advanced drug repurposing strategies. We conclude with a brief discussion of the human case studies using targeted pathway inhibitors for improved outcomes in individuals with CCLA.

## Advances in lymphatic system imaging enhance CCLA diagnosis

As the knowledge base about the flow and function of the lymphatic system grows, diagnostic options have improved with the development of more-effective methods for imaging the lymphatic system. Traditional MRI may visualize aspects of the lymphatic system such as the thoracic duct, but it fails to characterize flow and function of the central lymphatic vasculature (66). Some radiologists found success with these techniques, but only under very specific positional and clinical circumstances (67). However, the combination of contrasted MRI with angiography techniques has led to a new imaging technique for the study of the lymphatic system.

Dynamic contrast-enhanced magnetic resonance lymphangiography (DCMRL) is essential for the diagnosis of lymphatic conduction disorders (68). This allows for dedicated visualization of central lymphatic flow (63, 69–73). The feasibility of DCMRL was initially studied in a porcine model due to the structural homology of their central lymphatic system to that of humans (71, 74). These studies found no adverse effects of intradermal or intranodal injection of contrast with subsequent DCMRL imaging and were able to clearly visualize central lymphatic structures such as the thoracic duct (71, 74).

**Figure 2:**
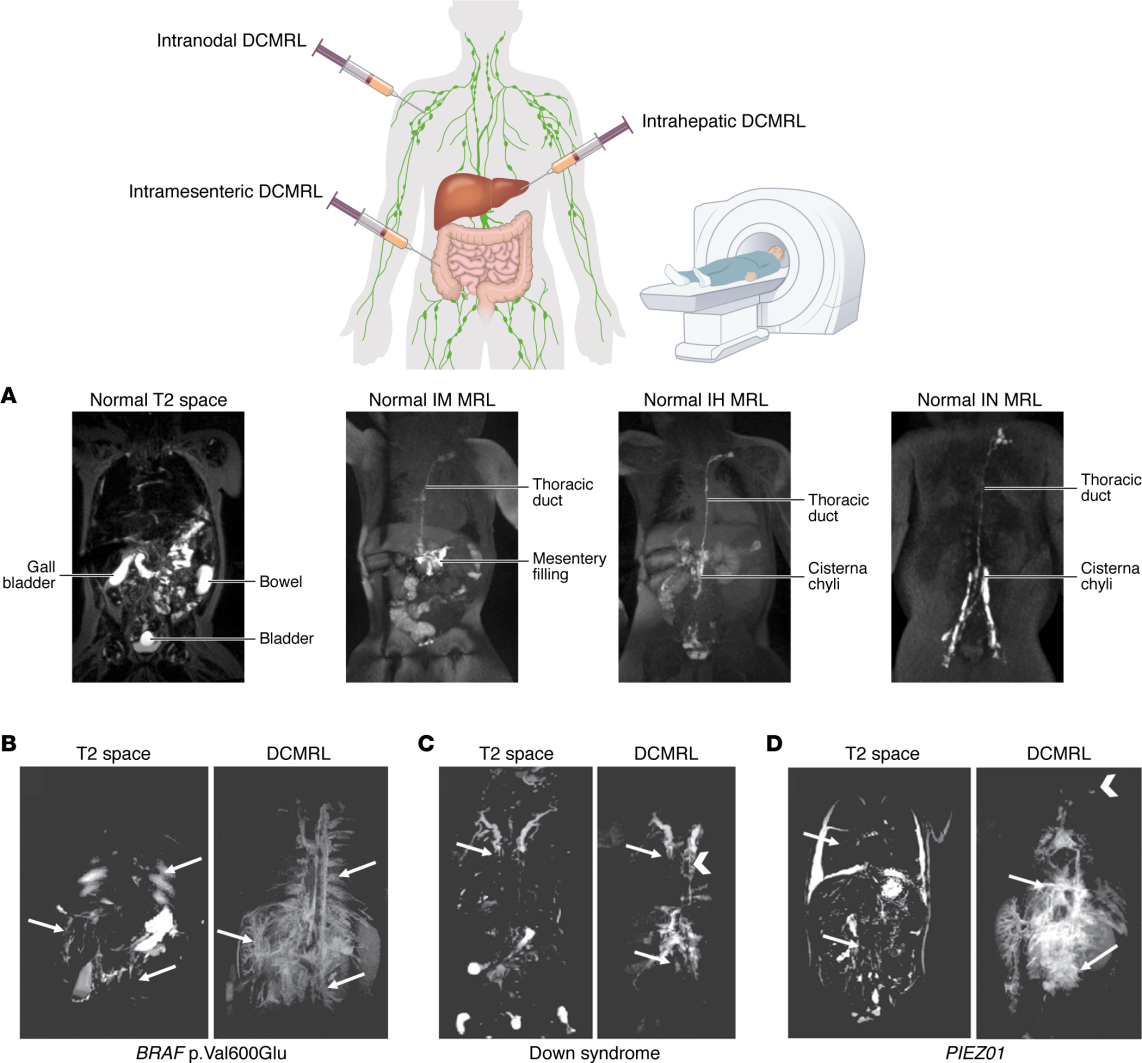
Schematic of the various DCMRL approaches. DCMRL can be performed using contrast that is injected into a lymph node, liver, and/or mesentery. Traditional T2 space MRI and DCMRL imaging representing the typical appearance of the lymphatic vessels (**A**) compared with their appearance in three individuals with CCLA due to mosaic *BRAF* (**B**), Down syndrome (**C**), and *PIEZO1*–generalized lymphatic dysplasia (**D**). In **B**, mosaic *BRAF* p.Val600Glu, T2 space imaging shows substantial edema in the intercostal, mesentery, and liver lymphatics that correlates with abnormal perfusion patterns on intrahepatic DCMRL. Also note the abnormal lymphatic thoracic vessels lacking a normal thoracic duct. In **C**, Down syndrome, T2 space imaging shows edema in the supraclavicular (and superior mediastinal) lymphatics (arrows). On intrahepatic DCMRL, there is retrograde flow into retroperitoneal lymphatics and intercostal, mediastinal, pulmonary, and supraclavicular perfusion (arrows). Arrowhead indicates a patent thoracic duct that courses to the left venous angle. In **D**, *PIEZO1*–generalized lymphatic dysplasia, T2 space imaging shows bilateral pleural effusions, and pulmonary and retroperitoneal edema (arrows). Intrahepatic DCMRL shows extensive flow to the hepatic capsular lymphatics, with extension into the mediastinum and pulmonary lymphatics (arrows). There is also retrograde flow into the retroperitoneal lumbar and mesenteric lymphatics. The arrowhead indicates a small thoracic duct coursing to the left venous angle, patent on follow-up imaging. IM, intramesenteric; IH, intrahepatic; IN, intranodal. Images reproduced from ref. 61 with permission.

The technique has been developed using three different approaches: intranodal (IN), intrahepatic (IH), and intramesenteric (IM) (Figure 2A). Researchers found that IN contrast injection allows for more-localized visualization of the lymphatic system and may be more appropriate in pediatric individuals compared with the more elaborate conventional lymphangiography (72, 75). For the investigation of IH lymphatic flow disorders, DCMRL was identified as an effective method for visualization of lymphatic flow in and around the liver, including the cisterna chyli (76). IM DCMRL was identified as a superior method for identifying peritoneal and duodenal leaks compared with the IN and IH approaches, indicating that it is more efficacious in the diagnosis and treatment of CCLA with these features (77). DCMRL can be performed from any lymph node access point to assist in the diagnosis of conduction issues, such as in one case study of an individual with GSD (78).

Evaluation of central lymphatic flow patterns demonstrated distinct features in individuals with various genetic disorders, such as RASopathies, Down syndrome (trisomy of chromosome 21), and *PIEZO1* generalized lymphatic dysplasia (Figure 2, B–D). RASopathies, which include Noonan syndrome, tend to have abnormalities of the thoracic duct and intercostal flow (61, 79–81). Individuals with Down syndrome have dilation of the lymphatics of the head and neck (61). Characteristic imaging findings in individuals with *PIEZO1*–generalized lymphatic dysplasia include connections between the hepatic and peribronchial lymphatics (61). Importantly, if genetic testing is negative or unavailable, the abnormal lymphatic flow patterns identified by DCMRL may hint at genetic diagnosis and possibly guide medical treatment. While not yet widely available, DCMRL is a useful tool in the diagnosis of CCLA and may facilitate interventions. The Lymphatic Education and Research Network maintains a list of Comprehensive Centers of Excellence (https://lymphaticnetwork.org/centers-of-excellence), many which offer DCMRL.

## State-of-the-art genetic advances facilitate identification of genetic causes

The advent of deep sequencing has allowed for identification of the genetic causes of CCLA. Notably, identification of the genetic cause can help refine clinical diagnosis and provide important information about the underlying pathophysiology as well as directing medical therapy. Genetic sequencing can be performed from either genomic DNA or cell-free DNA (cfDNA). Initial retrospective cohort studies demonstrated that about 25% of individuals with primary CCLA have a cause that can be identified from clinical genetic testing (61). More recent work suggests this rate may increase up to about 40% by use of a comprehensive sequencing approach and novel techniques (48). In secondary CCLA, usually no genetic cause is identified (48).

Genetic causes of CCLA may be germline or somatic. Germline genetic cause can easily be discovered from leukocyte genomic DNA isolated from either a saliva or blood sample. Somatic causes must be identified from an affected tissue source, which often can be complicated in CCLA. Recent work demonstrated that ultra-deep sequencing using unique molecular identifiers and cfDNA isolated from blood or lymphatic fluid can identify causative variants in CCLA (48). Clinically discarded samples, such as pleural fluid, were processed to enrich for endothelial cells using centrifugation, endothelial cell–specific cell culture conditions, and CD31 magnetic bead enrichment (48). These endothelial cells constituted an affected tissue sample that then was processed for sequencing.

These techniques have allowed researchers to identify single-gene disorders causing CCLA (Table 1). Mosaic and germline RASopathies due to pathogenic variants that result in upregulation of the RAS/MAPK signaling pathway are the most common causes of CCLA (Figure 1B) (48, 61, 80–88). Specifically, somatic pathogenic variants in *ARAF*, *BRAF*, *KRAS*, and *MAP2K1* resulting in isolated lymphatic as well as syndromic presentations have been identified (48, 61, 82, 85). Germline monoallelic pathogenic variants in *PTPN11*, *KRAS*, *HRAS*, *BRAF*, *RAF1*, *RIT1*, *SOS1*, *SOS2*, and *RASA1* have been identified in individuals with CCLA and Noonan syndrome, CCLA and Costello syndrome, CCLA and cardiofaciocutaneous syndrome, or CCLA and capillary malformation-arteriovenous malformation syndrome (48, 61, 80–88). Interestingly, the phenotypic heterogeneity may be due to second hits, as has been demonstrated in *RASA1* disorders (89–93). Other single-gene causes that have been described include *PIEZO1*, *FOXC2*, *EPHB4*, *MDFIC*, *JAG1*, *PIK3CA*, *GBA*, and *GBE1* (48, 61, 94–97). *GNA11* was identified as a potential candidate gene, but functional studies are needed (48).

Chromosomal disorders have also been identified in connection with CCLA (Table 1). The most common is Down syndrome, caused by the presence of an additional chromosome 21 (61). 22q11.2 deletion syndrome, previously known as velocardiofacial syndrome, has been identified in individuals with CCLA (61). A chromosome 4 duplication was previously described in an individual with CCLA; however, no somatic testing was performed (98).

These research advances and the body of knowledge that has grown about the genetic causes of CCLA suggest that all individuals with CCLA should undergo clinical genetic testing. Clinically available genetic diagnostic tests are gene panels, exome sequencing, and genome sequencing from genomic DNA isolated from leukocytes or other tissues (99). One limitation of gene panels is that they are only useful in the diagnosis of known genetic causes (100). Exome sequencing will identify variants in the protein-coding areas of DNA, whereas genome sequencing includes both coding and noncoding areas of DNA, though at lower coverage than exome sequencing. The methods chosen should be targeted to the individual’s characteristics. Clinical development of vascular anomaly–specific cfDNA and lymphatic fluid–based tests will provide a noninvasive method for somatic testing when affected tissue is not readily available for testing (48). Furthermore, evaluation of the utility of long-read genome sequencing may identify novel causes.

## Preclinical models enable personalized medicine approaches

**Figure 3:**
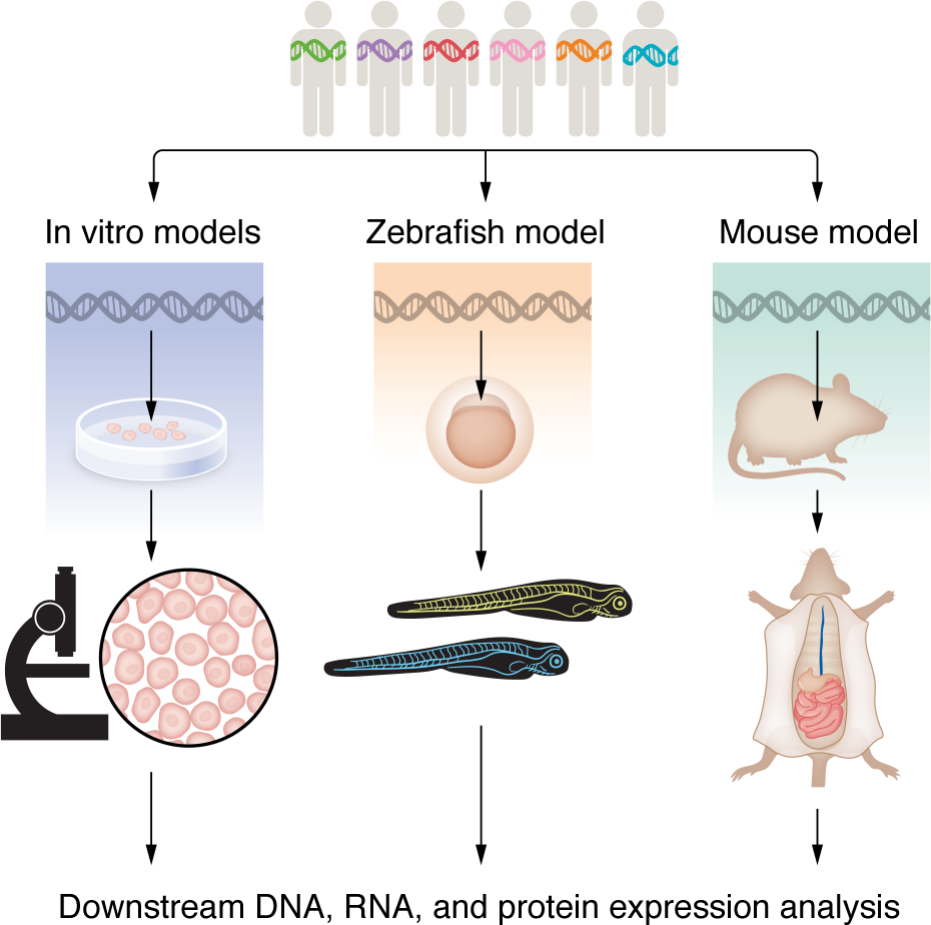
Overview of preclinical models of CCLA. Various in vitro models can be used to investigate cell-specific gene expression, endothelial migration, barrier formation, and cell proliferation. Utilization of the zebrafish to express affected individual–specific gene variants can be used for fluorescence imaging of the lymphatics and pharmaceutical screening. The murine model allows for a more in-depth tissue analysis of lymphatic vessels in mutant models of affected individual–specific variants and for targeted drug evaluation.

Investigation of the mechanisms underlying CCLA and validation of candidate genes hinges on effective in vitro and in vivo preclinical models to study lymphatic development and disease (Figure 3). In vitro models allow for efficient characterization of cellular behavior and molecular mechanisms (101). Vertebrate models such as zebrafish and mice can more effectively model physical anomalies and monitor phenotypes (101). Importantly, current models may help us identify novel human disease genes or validate candidate human disease genes. For rare diseases such as CCLA that do not have an effective treatment, these models can also allow for pharmaceutical safety and efficacy testing, leading to novel therapeutics for affected individuals.

### In vitro models of CCLA.

In vitro models using primary or derived human cell lines allow for more rapid investigation of the cellular and molecular mechanisms behind CCLA (Figure 3 and Table 2). Viral vectors allow for the transfection of aberrant proteins of interest into cell lines for downstream analysis of cellular mechanisms. Coupled with cell-specific assays, these allow for the investigation of the cellular mechanisms behind CCLA.

To investigate human-specific pathogenic variants in *MDFIC* (MyoD family inhibitor domain–containing protein), an autosomal recessive candidate gene for CCLA, researchers used primary adult human dermal LECs (HDLECs) and HEK293T cells (95). siRNA knockdown of MDFIC in HDLECs resulted in increased adhesion and decreased migration compared with controls. Ectopic expression of participants’ variants in HEK293T cells demonstrated truncation of the MDFIC p.M131fs* protein and that the truncated protein was not present at the cell surface. Furthermore, coexpression of MDFIC with GATA2 in HEK293T cells showed that the mutant protein did interact with GATA2, which plays an essential role in lymphatic development and maintenance. Overall, using a combination of different cells and traditional techniques such as immunoblotting, FACS, immunoprecipitation, adhesion, and migration assays, the researchers were able to define the cellular behavior of the mutant MDFIC.

**Figure 4:**
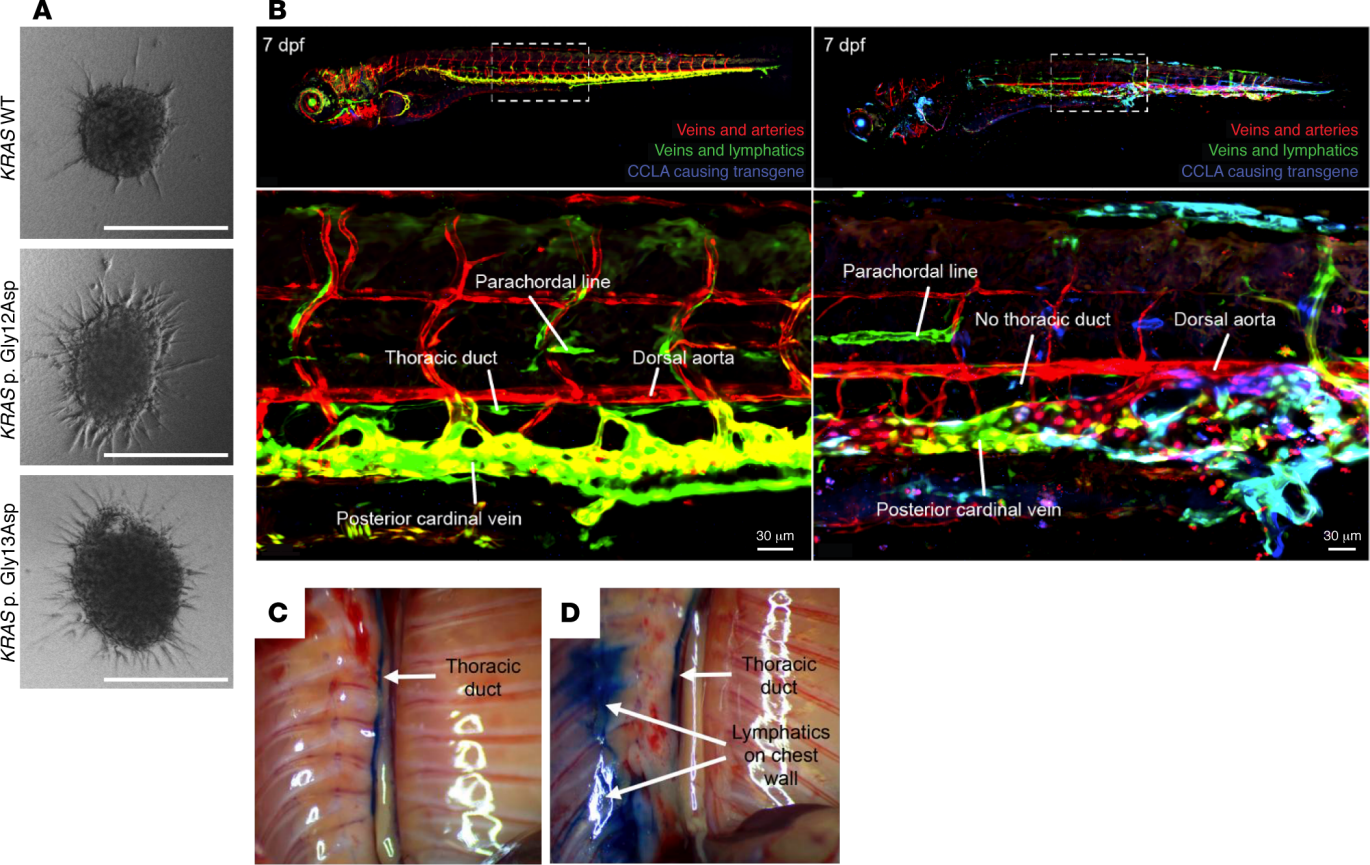
Examples of in vitro and in vivo assays to model CCLA. (**A**) In vitro organoid model. Lymphatic organoids were transduced with KRAS WT, KRAS p.Gly12Asp, or KRAS p.Gly13Asp and treated with DMSO (control); scale bars: 300 μm (images reproduced from ref. 85). (**B**) Zebrafish model of CCLA. Images of representative zebrafish 7 days post-fertilization (dpf) that were either uninjected or injected at the one-cell stage with a RASopathy-causing genetic variant under control of a lymphovenous (*mrc1a*) promoter. The top images show larvae that contain the Tg(*kdrl*:mCherry) transgene to label veins and arteries and Tg(*mrc1a*:EGFP) transgene to label veins and lymphatics. Additionally, the blue channel shows the mosaic expression of the *mrc1a*:RIT1:EBFP2 transgene, which causes profound lymphovenous malformations. The bottom images show the area within the dashed lines in the top images, labeled to highlight the morphological defects in the CCLA model fish. (**C** and **D**) Central lymphatics in iLEC^Kras^ mice were imaged on P20 with Evans blue dye. (**C**) The thoracic duct in iLEC^Ctrl^ mice (*n* = 4) filled with Evans blue dye (arrow). (**D**) The thoracic duct and lymphatics on the chest wall filled with Evans blue dye (arrows) in 4 of 6 iLEC^Kras^ mice. Images in **C** and **D** reproduced from ref. 24.

RASopathy-associated pathogenic variants result in cellular defects as well as increased lymphangiogenesis. To evaluate this, researchers transduced mutant proteins of interest into HDLECs, followed by immunostaining for cytoskeletal proteins or conducting a spheroid sprouting assay (48, 82, 85). Expression of mutant ARAF protein led to increased internalization of VE-cadherin, abnormal actin cytoskeleton, and elongation of the cells, which was improved by trametinib treatment. Similar results were seen with expression of mutant KRAS proteins. Spheroid models are also an effective three-dimensional method for evaluating the effect of a variant on lymphangiogenesis (Figure 4A) (48, 82, 85). These assays demonstrated increased sprouting behavior, including cumulative sprout length and number of sprouts per sphere in *ARAF*, *KRAS*, *BRAF*, and *RAF1* models, which was also reduced with trametinib (48, 82, 85). Additionally, lysates from spheroid models can be used in typical biochemical assays and demonstrated increased ERK phosphorylation at Thr202/Tyr204, which was inhibited by trametinib (48, 82, 85).

More-advanced techniques such as “organ on a chip” will allow for affected-individual specific cellular models that can mimic in vivo assays (102–104). Although previous work used isolated endothelial cells from individuals with CCLA for genomic analysis, some studies have isolated LECs from other lymphatic malformations and cultured these for downstream analysis (48, 105, 106). In combination with organ-on-a-chip models, this could facilitate investigations of mechanisms such as endothelial cell adhesion, membrane permeability, and fluid dynamics and personalized therapeutic approaches, benefitting individuals without an identifiable genetic cause (102–104).

### Zebrafish as a model for CCLA.

Zebrafish are a well-established model for developmental biology and have been an increasingly popular model organism of choice for vascular and lymphatic researchers since the characterization of the developing zebrafish lymphatic system (Figure 3) (6, 107–111). They are optically clear and externally fertilized, which allows for high-resolution analysis of developmental processes in living specimens. Augmenting the value of zebrafish in live imaging is the availability of an expansive collection of fluorescent marker lines, making imaging and identification of developing structures much easier (27, 110, 112, 113). Additionally, zebrafish yield fantastic opportunities for molecular investigation because they are highly manipulatable, have excellent genetic conservation, and have comparatively high fecundity, which allows for rapid and high-throughput pharmaceutical screening. Finally, zebrafish have striking morphological similarity to humans, including the presence of the thoracic duct, which models the thoracic duct in humans. With these characteristics taken together, zebrafish are an effective model to examine phenotype and developmental changes.

There are multiple methods available to model specific variants in the zebrafish (113). Loss-of-function variants can be modeled using CRISPR/Cas9 to decrease in the activity of the protein of interest. Although morpholinos (MOs) are still occasionally used for gene knockdown, mutant phenotypes may not be congruent with MO phenotypes (114). Gain-of-function variants can be expressed throughout the embryo or in a cell type–specific manner by either mRNA injection or the Tol2 transposase system (115, 116). Following a bedside-to-bench or forward-genetics workflow, pathogenic variants or variants of uncertain significance can first be identified in individuals affected by CCLA. Once a gene of interest is identified, researchers can choose the appropriate tool and create a zebrafish to model an affected individual. The utility of the zebrafish model goes beyond modeling phenotypical characteristics. As a result of the zebrafish’s highly conserved molecular pathways, downstream molecular analyses performed by isolating macromolecules from mutant embryos — including typical techniques such as quantitative real-time PCR (qRT-PCR), RNA-Seq, and Western blotting — can also be utilized to continue to investigate the underlying pathways of CCLA.

A proof-of-principle example has already been demonstrated, wherein a splice site *EPHB4* pathogenic variant was identified in an individual with CCLA and later modeled in Tg(*fli1*:EGFP) zebrafish using MO to knock down *ephb4* gene expression (Table 3) (94). The Tg(*fli1*:EGFP) zebrafish line enables researchers to visualized blood and lymphatic vessel formation during development (117). The model displayed misbranching of the intersegmental vessels and cystic formation in the caudal plexus. Treatment with mTORC1, MEK1/MEK2, or PI3K/mTOR inhibitors led to a significant reduction in misbranching and cyst formation, confirming that both pathways are important for the development of this phenotype. However, as in other applications, MO may induce off-target p53 effects and is diluted over time, which reduces their efficacy, and thus is not conducive to long-term examination (114).

Pathogenic variants in *ARAF*, *RIT1*, and *KRAS* were identified in individuals with CCLA (Table 1) (82, 85). Using the lymphatic- and venous-specific promotor *mrc1a* with Tol2-mediated transgenesis, researchers discovered that expression of these pathogenic variants led to pericardial edema and dilation of the thoracic duct (Table 3). In the ARAF-mutant model, treatment with cobimetinib, a MEK1/MEK2 inhibitor, resulted in significantly fewer larvae with severe dilation of the thoracic duct (82). Interestingly, in the KRAS-mutant models, there were genotype-specific differences. Treatment with sirolimus, an mTORC1 inhibitor, resulted in a significant reduction in the fraction of larvae with pericardial edema in the p.G13D but not the p.G12D model (85). In ongoing studies, approaches similar to those used for *KRAS* are being used to investigate pathogenic *RIT1* variants, with similar results (Figure 4B). These results suggest that some pathway inhibitors may be efficacious for RASopathies; however, there may be nuances in therapy based on genotype that still must be investigated. Overall, the benefits of the zebrafish model allow for quick, personalized-medicine approaches for investigating therapies.

### Mice as a model for CCLA.

Mice offer several benefits in biomedical research, including their phylogenetic and physiological similarity to humans, the ease of breeding and housing in a laboratory setting, and the commercial availability of inbred strains. One of the most powerful advances offered by this model is the rich toolbox of existing genetic tools including Cre/loxP recombination, tetracycline-inducible expression, CRISPR/Cas9 gene editing, Gateway transgenic recombination, and blastocyst chimeric injections (118). Genetic tools are used to induce expression of a certain gene or pathogenic variant in the specific cell populations or create a mutation in a mouse gene to produce a nonfunctional protein or completely prevent translation, called “knockin” or “knockout,” lines respectively. Mice have proven to be an ideal system for studying vascular development on multiple levels, from LEC proliferation to postnatal vessel branching and vascular-disease progression through adulthood (3). Utilizing the previously mentioned techniques, researchers can genetically manipulate a certain target gene and then characterize vascular development to understand the pathway by which a certain genetic target affects lymphatic biology. Like DCMRL in humans, dynamic imaging can be performed to evaluate the structural and functional competence of lymphatic vessels by revealing the flow of fluorescent-labeled tracers or Evans blue dye through the thoracic duct and adjacent lymphatics in mice (Figure 4, C and D) (24).

Currently, there are few published mouse models for CCLA (Table 4). Using CRISPR/Cas9, researchers generated a loss-of-function mouse model to evaluate the involvement of *MDFIC* after biallelic variants were found in seven individuals affected by CCLA (95). Like the affected individuals, *Mdfic^M131fs/M131fs^* homozygous mice had congenital chylothorax (95). They also demonstrated dysmorphic thoracic duct and intercostal lymphatics with retrograde lymph flow, increased amounts of LYVE1-positive macrophages, and defective lymphatic valves, which resulted in total perinatal lethality by 30 days (95). RASA1 is a GTPase-activating regulator of blood and lymphatic vessel development and is an autosomal dominant cause of capillary malformation–arteriovenous malformation syndrome (CM-AVM) and CCLA in humans (119). Like humans, mice lacking *Rasa1* had extensive lymphatic vessel hyperplasia and leakage with chylothorax likely due to the loss of lymphatic endothelial cells in the valve leaflet (120–122). A RASA1 inducible-deletion model showed constitutive activation of Ras that led to LEC proliferation and lymphatic hyperplasia, which could be prevented through blocking the upstream growth factor receptor VEGFR-3 (121).

Other mouse models recapitulate other complex lymphatic anomalies and serve as a valuable tool for therapeutic screening in a bench-to-bedside model. A mouse model of GLA used the lymphatic vessel–specific Prox1-CreER^T2^ inducible mouse and LSL-Pik3ca^H1047R^ mice to drive expression of the p.His1047Arg somatic pathogenic *Pik3ca* variant in LECs expressing the fluorescent marker GFP (123). Rapamycin was shown to both prevent and improve progressive lymphatic vessel disease in mice and reduce pain and functionality in humans (123). *PIK3CA^VEGFR3-CreER^* mice expressed a constitutively active p110 protein chimera and developed phenotypes similar to those of humans, including vessel lymphatic malformations, chylothorax-associated lesions, gastrointestinal anomalies, and leaky vessels (124). Treatment with alpelisib (20 mg/kg), a PIK3CA inhibitor, starting on the last day of Cre induction rescued *PIK3CA^VEGFR3-CreER^* mice by restoring normal lymphatic vascular distribution and integrity by 30 days after induction and reducing the size of existing lymphatic malformations by an average of 90.6% (124). Gorham-Stout disease (GSD) has also been studied in the *iLEC^KRAS^* mouse model using Prox1-CreER^T2^ to drive LEC-specific expression of KRAS (G12D), after an activating somatic variant in *KRAS* (G12V) was identified in one individual with GSD (24). The *iLEC^KRAS^* model recapitulated phenotypes including the development of ectopic lymphatics in bone tissue; and changes to vessel structure, including lymphatic valve regression, which was shown to be prevented by treatment with trametinib from P0 to P12 (24). Interestingly, *iLEC^KRAS^* mice showed abnormal posterior intercostal flow with IN injection, similar to the abnormal flow visualized by DCMRLs in humans with RASopathies (24, 61, 79–81).

Pathogenic variants in connexin genes cause lymphatic disease as a part of various syndromes in humans, and although CCLA has not been reported in these syndromes, evidence linking connexins to CCLA has been reported in mice. Variants in CX43, encoded by *GJA1*, and CX47, encoded by *GJC2*, cause lymphedema in humans, which can be a clinical manifestation of CCLA (125–127). In mice, loss of CX43 and CX37 led to lethal chylothorax, lymphedema, and bloody lymphatic vessels in the intestine and skin despite normal blood vasculature structure, whereas the lymphatic-specific ablation of CX43 resulted in a delay in lymphatic vessel formation — with fewer lymphatic valves that were immature and leaky due to incomplete leaflet elongation — but also lethal chylothorax (128, 129). Further research demonstrated significant valve defects; and evaluation of pressure back-leak in vessels through quantification of vessel dilation and use of the servo-null micropressure system demonstrated the valve leaflet regression and valve dysfunction observed in *Cx43*- and *Cx37*-mutant mice (130, 131).

Across the past two decades, researchers have used these genetic techniques to identify proteins important in lymphatic development that likely will be discovered as causes of CCLA. A novel adipocyte-specific VEGF-C overexpression model recapitulated the enlarged lymphatic vasculature and leakage phenotypes observed in human chylothorax patients when visualized with rhodamine-labeled *Ricinus communis* agglutinin I (RCA I) lectin tracer injection (132). α_9_β_1_ Integrin is a receptor for extracellular adhesion proteins, such as osteopontin, tenascin-C, and vascular cell–specific immunoglobin adhesion molecules (133). Homozygous null mutants lacking the α_9_ subunit developed edema and lymphatic vessel infiltration of the chest wall that ultimately led to death within 1–2 weeks due to accumulation of fluid in the lungs (133). A hypomorphic mutant lacking the NET DNA–binding domain, a serum response element repressor of vasculogenesis, showed vascular defects including lymphatic vessel dilation and chylothorax-induced respiratory failure (134). Loss of NET was associated with upregulation of EGR-1, a transcription factor known to be a downstream target of ERK and AKT signaling and activator of VEGF-A (134). Endothelial cell MAP4K4 has been shown to be essential to lymphatic vascular development by use of an endothelial cell–specific Cre-inducible loss-of-function mutant model (135). MAP4K4-deficient mice to develop lethal postnatal chylothorax in addition to lymphatic capillary dilation, reduced valve numbers in the collecting lymphatics, and impaired lymphatic flow (135).

Mouse models have proven to be a fruitful tool for expanding the currently limited body of research on lymphatic anomalies, including phenotypic modeling and development of targeted therapies. Additional techniques to isolate the endothelial cells from mouse collecting vessels in combination with in vitro systems can act in concert with mouse models to provide a deeper insight into the basic biology of lymphatic development and disease (136).

## Targeted medical therapy for CCLA

The discovery of effective therapeutics in in vitro and in vivo models of CCLA have been translated to medical therapy for humans (Table 5). While early studies focused on using the mTOR inhibitor sirolimus (also known as rapamycin) agnostic of genetic cause, more recent studies have used other pathway inhibitors targeted to the molecular underpinnings of the disease.

Early trials focused on the use of sirolimus, an mTOR inhibitor, in vascular anomalies. The initial trial included only three participants with CCLA, who all had progressive disease while on treatment (137). However, an individual with CCLA had a reduction in chylous drainage after starting sirolimus, and another retrospective cohort study of infants with chylothorax due to GLA or CCLA found a reduction in mean duration with chest tube compared with prior studies (138, 139). As CCLA due to *PIK3CA* is rarer, there is less information about the use of alpelisib for CCLA. In one individual with features of CCLA and GLA due to a somatic pathogenic variant *PIK3CA*, there was reported improvement in lymphatic system function, as visualized by DCMRL, after initiation of alpelisib (48). There is crosstalk between the RAS and PI3K pathways, so in the future additional animal models or treatment trials may focus on combined therapy.

Given that the most common genetic causes of CCLA are activating variants in the RAS pathway, it is not surprising that initial studies targeting the RAS pathway demonstrated efficacy. The initial studies in zebrafish modeling *ARAF* variants paved the way for trialing therapy with the MEK inhibitor trametinib in a single case study (82). This individual affected with CCLA and lymphedema due to *ARAF* had improvement on pulmonary function tests, reduction in lymphatic fluid retention, decrease in supplemental oxygen requirements, and altered flow as visualized by DCMRL (82). Subsequently, trametinib was used to treat an individual with Noonan syndrome caused by a *SOS1* variant (*SOS1*–Noonan syndrome) and CCLA manifesting as protein-losing enteropathy (83). Trametinib treatment resulted in normalization of albumin level within 3 months of therapy (83). In an infant with *SOS1*–Noonan syndrome and pulmonary lymphangiectasia and pleural effusions requiring invasive ventilation, trametinib treatment resulted in resolution of the effusions within 1 week and transition to room air after 3 weeks (140). In an adult with *RIT1*–Noonan syndrome and lymphatic failure, trametinib therapy resulted in increased albumin level, resolution of ascites in 3 months, and reduction of pericardial effusion within 18 months (84). Similarly, in a child with *RIT1*–Noonan syndrome who had chylous pleural effusions drained at the initiation of a 3-month course of trametinib, the effusions did not recur (141). In another case series, three infants with Noonan syndrome (*SOS1*, *RIT1*, *PTPN11*) all had a reduction in chylous effusions, chylous ascites, and improvement in respiratory status with trametinib therapy (86). Although many of these single cases show success, there have been no clinical trials yet to truly assess the safety and efficacy of trametinib or other MEK inhibitors in the treatment of CCLA.

Therapies targeted to an individual’s dysregulated molecular pathway in combination with conservative management have resulted in improved outcomes for individuals with CCLA. These medicines may obviate the need for more-traditional management or may be combined with conservative management for the best outcome.

## Conclusion

CCLA is a complex lymphatic anomaly that has historically been difficult to diagnose and treat. Advances in imaging and genomics have improved diagnosis and refined understanding of both genotype and phenotype. Further clinical development of liquid biopsy will allow for noninvasive genetic diagnosis. In vitro and in vivo models have validated various candidate genes that cause CCLA and defined the underlying pathogenesis. These affected individual–based models facilitated development of personalized therapeutics targeted to genetic cause, resulting in improved outcomes. Continued collaboration between basic scientists and clinicians will continue to drive forward the science to understand the molecular underpinnings of CCLA, visualize the lymphatic system, and identify pharmaceutical treatments to improve outcomes in individuals with CCLA.

## Figures and Tables

**Table 5:**
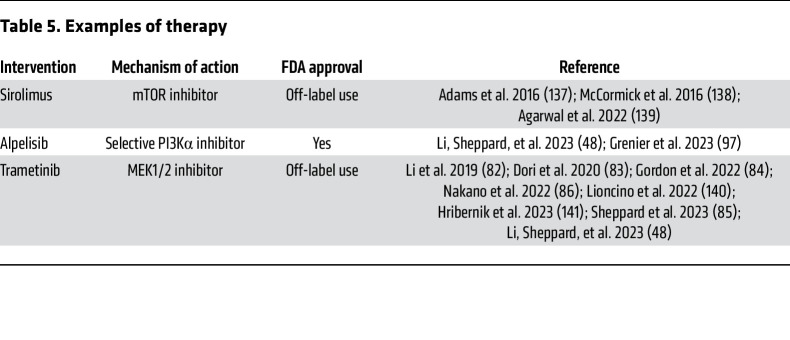
Examples of therapy

**Table 4:**
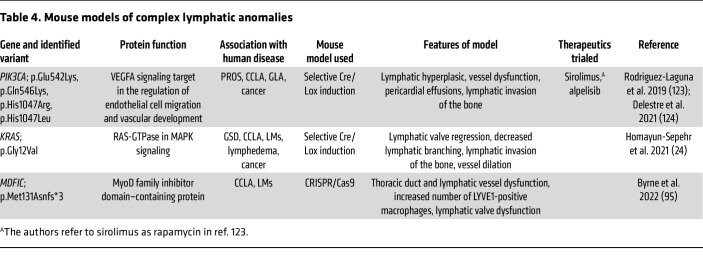
Mouse models of complex lymphatic anomalies

**Table 3:**
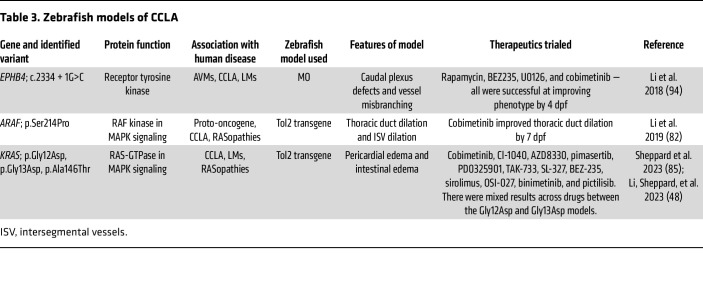
Zebrafish models of CCLA

**Table 2:**
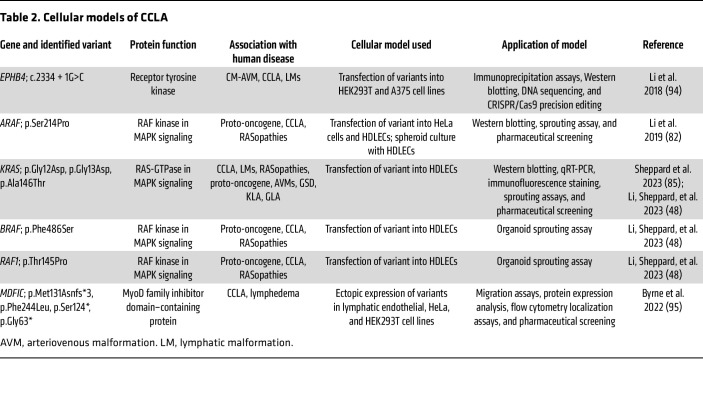
Cellular models of CCLA

**Table 1:**
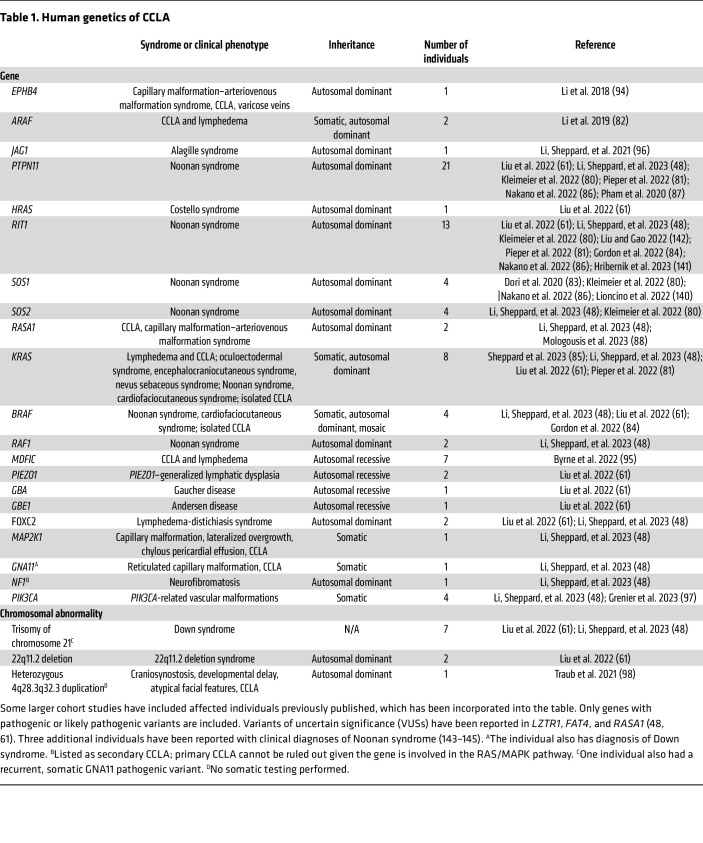
Human genetics of CCLA

## Version 1 04/15/2024

Electronic publication
